# Supplementary material for: Caenorhabditis elegans LET-381 and DMD-4 control development of the mesodermal HMC endothelial cell
Source: Development. 2025 Jul 29;152(14):dev204622. doi: 10.1242/dev.204622 (PMC12377808; doi:10.1242/dev.204622)
Supplement: Supplementary information [file develop-152-204622-s1.pdf]

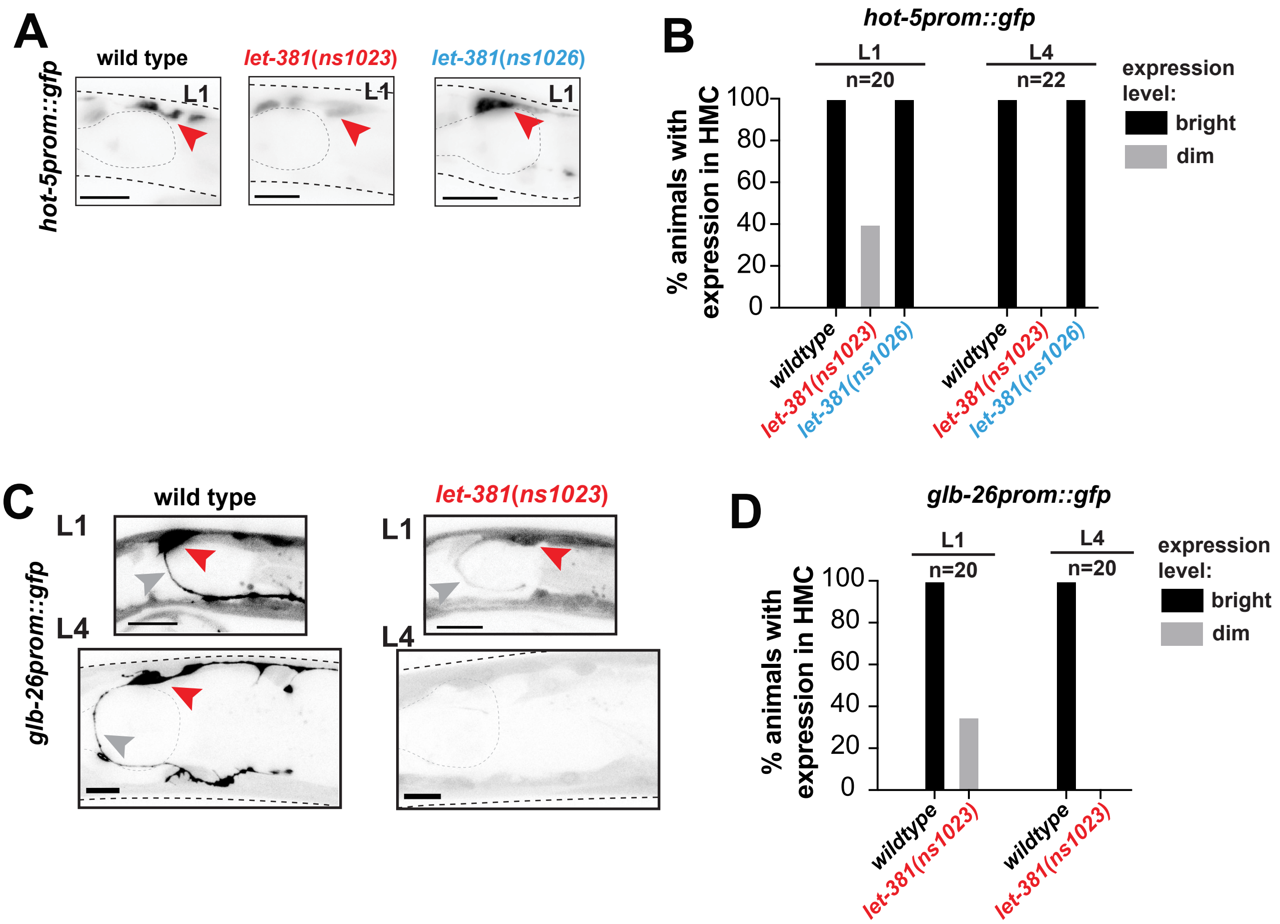

Fig. S1. Postembryonic LET-381 downregulation, *let-381(ns1023)*, in the HMC cell, affects maintenance and not initiation of HMC gene expression.

(A-D) *hot-5prom::gfp* and *glb-26prom::gfp* expression in HMC is gradually lost in *let-381(ns1023)* mutant animals. (A) *hot-5prom::gfp* expression in wild-type, *let-381(ns1023)* and *let-381 (ns1026)* L1 larvae. Images of L4 animals are shown in Fig 3A. (B) Percentage of animals with *hot-5prom::gfp* expression in the HMC, in L1 and L4 larva stages. (C) *glb-26prom::gfp* expression in wild-type and *let-381(ns1023)* L1 (top) and L4 larvae (bottom). (D) Percentage of animals with *glb-26prom::gfp* expression in the HMC, in L1 and L4 larva stages. Red arrowhead: HMC. Grey arrowhead: HMC lateral process. Anterior is left, dorsal is up. Scale bars: 10 µm.

Development • Supplementary information

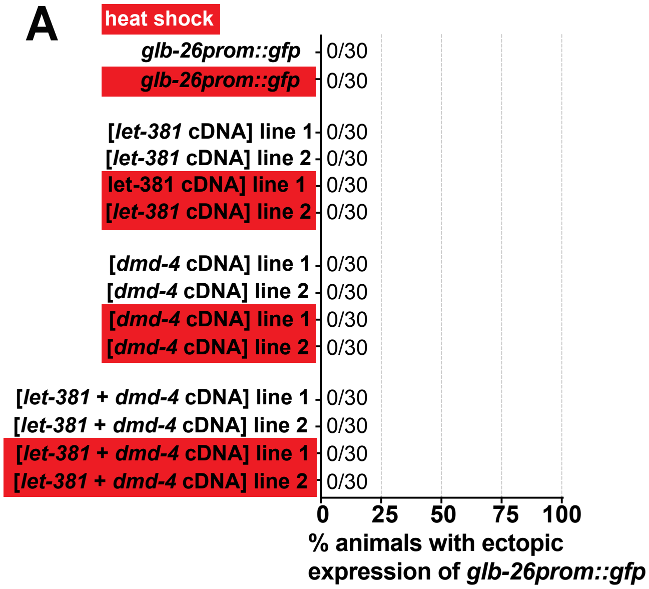

Fig. S2. LET-381 and DMD-4 are not sufficient to induce HMC gene expression in other cells.

(A) Percentage of animals with ectopic expression of the HMC reporter *glb-26prom::gfp*, upon heat shock induced misexpression of *let-381* (*hsp-16.2prom::let-381^{cDNA}*), *dmd-4* (*hsp-16.2prom::dmd-4^{cDNA}*) and both transcription factors together (*hsp-16.2prom::let-381^{cDNA}* + *hsp-16.2prom::dmd-4^{cDNA}*). Heat shocked animals (red boxes) are compared to age matched non-heat shocked controls. n=30 animals, for each genotype and condition.

Development • Supplementary information

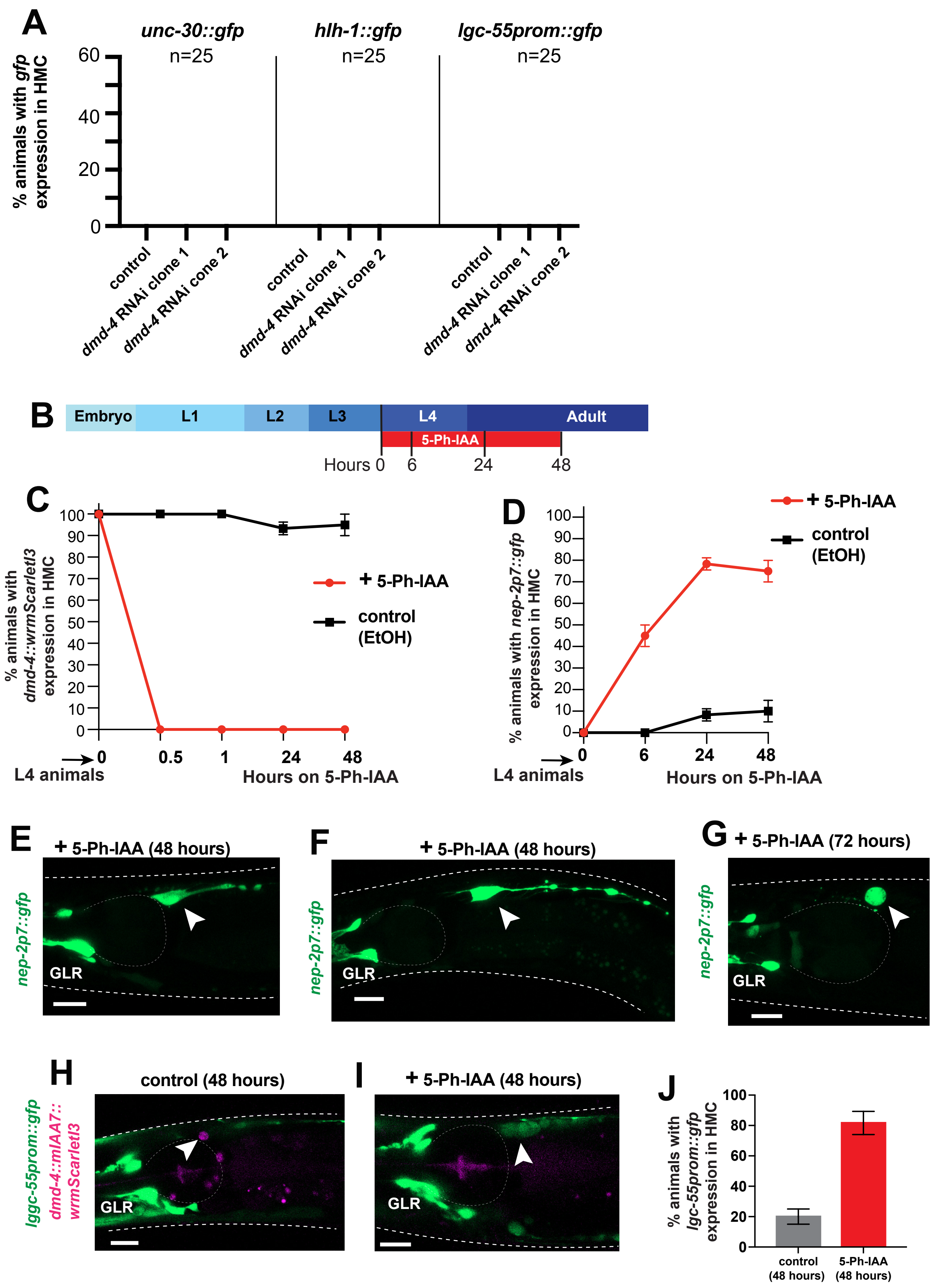
A
unc-30::gfp
n=25
hlh-1::gfp
n=25
lgc-55prom::gfp
n=25
% animals with gfp expression in HMC
60
40
20
0
control
dmd-4 RNAi clone 1
dmd-4 RNAi cone 2
B
Embryo
L1
L2
L3
L4
Adult
5-Ph-IAA
Hours 0
6
24
48
C
% animals with dmd-4::wrmScarlet3 expression in HMC
+ 5-Ph-IAA
control (EtOH)
0
0.5
1
24
48
L4 animals
Hours on 5-Ph-IAA
D
% animals with nep-2p7::gfp expression in HMC
+ 5-Ph-IAA
control (EtOH)
0
6
24
48
L4 animals
Hours on 5-Ph-IAA
E
+ 5-Ph-IAA (48 hours)
nep-2p7::gfp
GLR
F
+ 5-Ph-IAA (48 hours)
nep-2p7::gfp
GLR
G
+ 5-Ph-IAA (72 hours)
nep-2p7::gfp
GLR
H
control (48 hours)
lggc-55prom::gfp
dmd-4::mIAA7::wrmScarlet3
GLR
I
+ 5-Ph-IAA (48 hours)
GLR
J
% animals with lgc-55prom::gfp expression in HMC
100
80
60
40
20
0
control (48 hours)
5-Ph-IAA (48 hours)

Fig. S3. DMD-4 is required for repression of GLR glia genes in the HMC.
(A) Quantification of animals with ectopic HMC expression of three GLR glia reporters. None of these reporters is ectopically expressed in HMC upon *dmd-4*(RNAi). Number of animals (n) tested for each genotype for each reporter is shown under the name of each reporter transgene. (B) Timeline of DMD-4 auxin inducible degradation for L4 stage animals (L4 stage DMD-4 AID), showing time points relevant for C and D. (C) Quantification of DMD-4 degradation, based on wrmScarletI3 expression in HMC, at different time points for L4 stage DMD-4 AID. (D) Quantification of animals with ectopic HMC expression of the GLR-specific reporter *nep-2prom7*::*gfp* upon L4 stage DMD-4 AID at different time points. (E-G) Representative images showing *nep-2p7::gfp* in 5-Ph-IAA treated animals at different time points. White arrowheads, HMC. (H, I) Representative images showing *dmd-4::mIAA7::wrmScarletI3::mIAA7* (magenta) and *lgc-55prom::gfp* (green) for control and 5-Ph-IAA treated animals. White arrowheads, HMC. *lgc- 55prom::gfp* is ectopically expressed in HMC cells upon DMD-4 AID. (J) Quantification of percentage of animals with *lgc-55prom::gfp* expression in HMC after 48 hours on 5-Ph-IAA. For C, D and J three replicate experiments were performed, with n=20 animals per replicate for each condition. Error bars show standard deviation between replicates. Anterior is left and dorsal is up. Scale bars: 10μm.

CAGTGTCGACTCAATCTCTCCAAACTCGTCAACATTCGAATCGTCAGGCAGCGGTG
GCAGTGGAGGTACCGGCGGAAGCGGTGGATTCTCCGAGACCGTCGACCTCATGCT
CAACCTCCAATCCAACAAGGAGGGATCCGTCGACCTCAAGAACGTCTCCGCCGTC
CCAAAGGAGAAGACCACCCTCAAGGTAAGTTTAAACATATATATACTAACTAACCCT
GATTATTTAAATTTTCAGGACCCATCCAAGCCACCAGCCAAGGCCCAAGTCGTCGG
ATGGCCACCAGTCCGTAACTACCGTAAGAACATGATGACCCAACAAAAGACCTCCT
CCGACTCCACCGAGGCCGTCATCAAGGAGTTCATGCGTTTCAAGGTCCACATGGA
GGGATCCATGAACGGACACGAGTTCGAGATCGAGGGAGAGGGAGAGGGACGTCC
ATACGAGGGAACCCAAACCGCCAAGCTCAAGGTCACCAAGGGAGGACCACTCCC
ATTCTCCTGGGACATCCTCTCCCCACAATTCATGTACGGATCCCGTGCCTTCATCAA
GCACCCAGCCGACATCCCAGACTACTGGAAGCAATCCTTCCCAGAGGGATTCAAG
TGGGAGCGTGTCATGATCTTCGAGGACGGAGGAACCGTCTCCGTCACCCAAGACA
CCTCCCTCGAGGACGGAACCCTCATCTACAAGGTCAAGCTCCGTGGAGGAAACTT
CCCACCAGACGGACCAGTCATGCAAAAGCGTACCATGGGATGGGAGGCCTCCACC
GAGCGTCTCTACCCAGAGGACGTCGTCCTCAAGGGAGACATCAAGATGGCCCTCC
GTCTCAAGGACGGAGGACGTTACCTCGCCGACTTCAAGACCACCTACAAGGCCAA
GAAGCCAGTCCAAATGCCAGGAGCCTTCAACATCGACCGTAAGCTCGACATCACC
TCCCACAACGAGGACTACACCGTCGTCGAGCAATACGAGCGTTCCGTCGCCCGTC
ACTCCACCGGAGGATCCGGAGGATCCGGATTCTCGGAGACGGTCGATCTCATGCT
CAATCTCCAATCCAACAAAGAGGGATCCGTAGACCTCAAGAATGTCTCCGCCGTCC
CTAAGGAGAAGACGACCCTCAAGGATCCATCCAAGCCACCTGCCAAGGCCCAAGT
TGTCGGATGGCCACCAGTTCGTAACTACCGTAAGAATATGATGACGCAGCAGAAAA
CGTCGTCGTAATAAATTTATCTTTTTACACTTATCACGTGTTTTCAACCGTATTACTTG
TGGCAAATCCT

GREEN: **homology arms**
GREY: **linker**
YELLOW: **mIAA7**
RED: **wrmScarletI3**
PURPLE: **mutation PAM site from CAAA to TTTT**

Fig. S4. Sequence details of the Repair Template used for *dmd-4::linker::mIAA7::wrmScarletI3::mIAA7* endogenous tagging.

Development • Supplementary information

Table S1. List of strains used and generated in this study.

Strain Name	Genotype	Reference
N2	wild type	Caenorhabditic Genetics Center (CGC)
VC706	*let-381(gk302) I / hT2 (I;III)*	(Barstead et al., 2012)
KR429	*dpy-5(e61) let-381(h107) unc-13(e450) I ; sDp2 (I;f)*	(Howell et al., 1987)
GR1373	*eri-1(mg366) IV*	(Kennedy et al., 2004)
DE60	*dnIs13 [gly-18prom::gfp + unc-119(+)] I ; unc-119(e2498) III*	(Warren et al., 2001)
MT20492	*lin-15B&lin-15A(n765) X ; nIs471 [lgc-55prom::gfp + lin-15(+)]*	(Ringstad et al., 2009)
OH13027	*otIs569 [snf-11(fosmid)::SL2::H2B::mChopti + pha-1(+)]*	(Gendrel et al., 2016)
PD4443	*ccIs4443 [arg-1prom::gfp + dpy-20(+)] IV*	(Kostas and Fire, 2002)
SD1633	*ccIs4251 [(pSAK2) myo-3p::GFP::LacZ::NLS + (pSAK4) myo-3p::mitochondrial GFP + dpy-20(+)] I ; stIs10539 [dmd-4p::HIS-24::mCherry + unc-119(+)]*	(Liu et al., 2009)
OH16286	*dmd-4(ot933) X*	(Bayer et al., 2020)
HS3750	*ieSI58 IV ; osIs182 V*	(Negishi et al., 2021)
OS11703	*nsIs746 [nep-2prom7::gfp] V*	(Stefanakis et al., 2024)
OS12099	*nsIs831 [pll-1prom1::tagrfp] X*	(Stefanakis et al., 2024)
OS12103	*nsIs835 [hot-5prom2::gfp] X*	(Stefanakis et al., 2024)
OS12700	*unc-30(ns959[unc-30::gfp:::degron(AID)]) IV*	(Stefanakis et al., 2024)
OS13288	*let-381(ns995[let-381::gfp::aid]) I*	(Stefanakis et al., 2024)
PHX3025	*hlh-1(syb3025[hlh-1::gfp::aid]) II*	(Stefanakis et al., 2024)
PHX5792	*pll-1(syb5792[pll-1::sl2::gfp::h2b]) III*	(Stefanakis et al., 2024)
OS14214	*pll-1(ns1040[*syb5792]) III*	(Stefanakis et al., 2024)
PHX5759	*gbb-2(syb5759[gbb-2::sl2::gfp::h2b]) IV*	(Stefanakis et al., 2024)
OS13835	*let-381(ns1023[*ns995]) I*	This study
OS13838	*let-381(ns1026[*ns995]) I*	(Stefanakis et al., 2024)
OS15370	*dmd-4(ns1103[dmd-4::linker::mIAA7::::wrmScarletI3::mIAA7]) X*	This study
OS15371	*nsIs1052 [glb-26p::gfp , myo-3::mCherry]*	This study
OS15375	*pll-1(ns1104[*syb5792]) III*	This study
OS14217	*gbb-2(ns1043[*syb5759)] IV*	This study
OS15376	*nsEx7466 [hsp-16.2p::let-381 cDNA, pha-1+] ; pha-1(e2123) ; nsIs1052*	This study
OS15377	*nsEx7467 [hsp-16.2p::let-381 cDNA, pha-1+] ; pha-1(e2123) ; nsIs1052*	This study
OS15378	*nsEx7468 [hsp-16.2p::dmd-4 cDNA, pha-1+] ; pha-1(e2123) ; nsIs1052*	This study
OS15379	*nsEx7469 [hsp-16.2p::dmd-4 cDNA, pha-1+] ; pha-1(e2123) ; nsIs1052*	This study
OS15380	*nsEx7470 [hsp-16.2p::dmd-4 cDNA, pha-1+] ; pha-1(e2123) ; nsIs1052*	This study
OS15381	*nsEx7471 [hsp-16.2p::dmd-4 cDNA, pha-1+] ; pha-1(e2123) ; nsIs1052*	This study

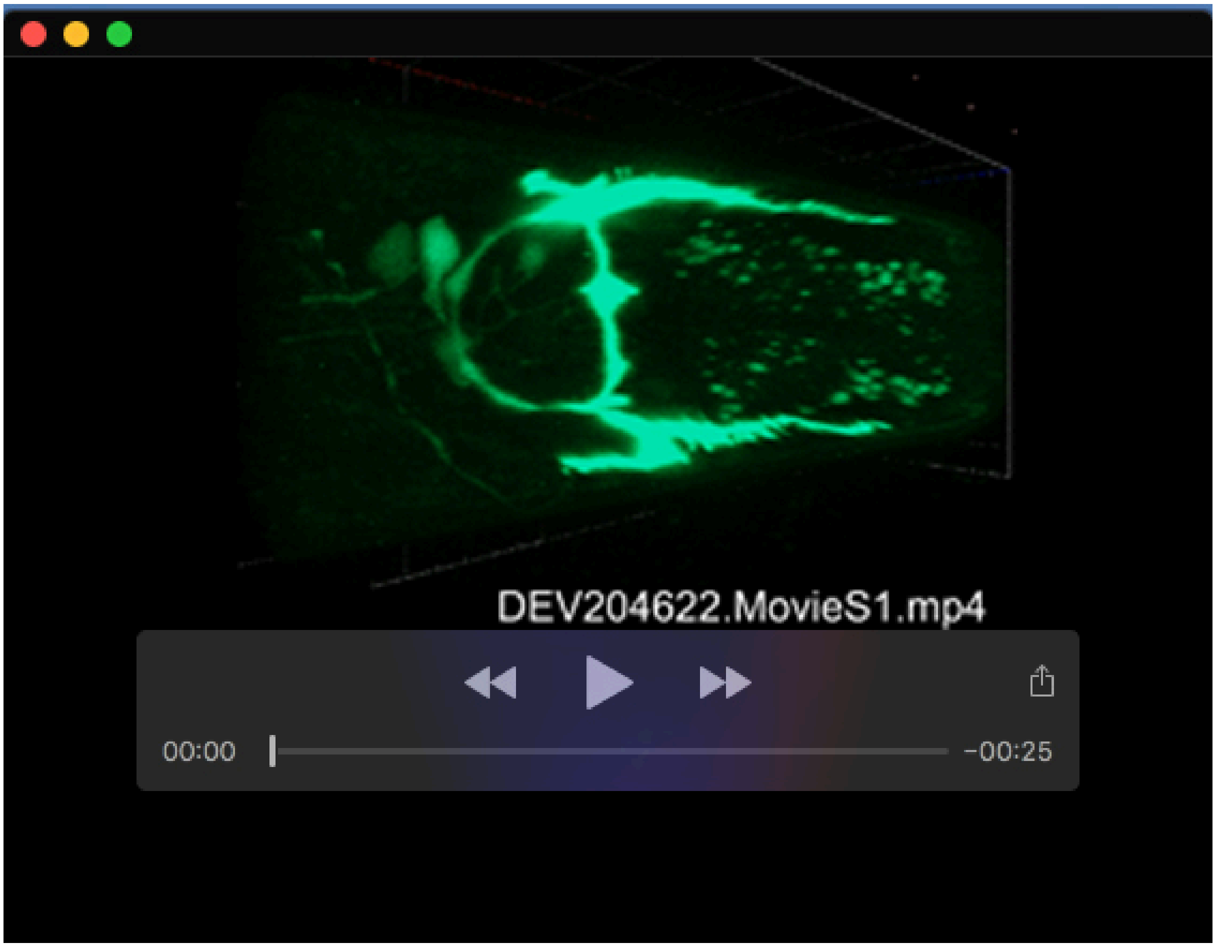

Movie 1. Wild type HMC morphology. *arg-1prom::gfp* expression in wildtype background. The HMC is the bright cell in the middle, dim *gfp* expression is observed in some neurons anterior (left) of the HMC.

Development • Supplementary information

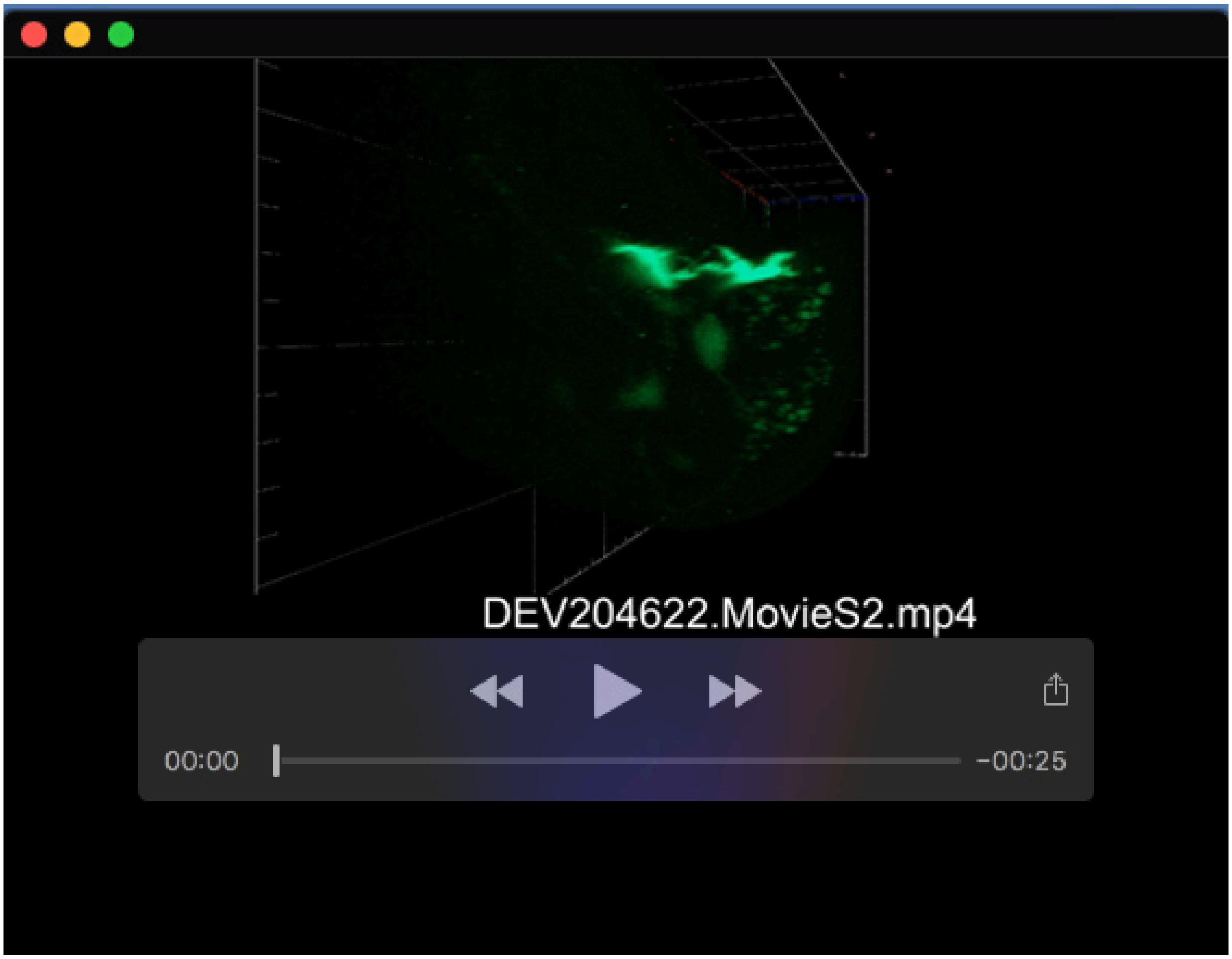

Movie 2. HMC with Defective Morphology in let-381(ns1023) mutant background, example 1. *arg-1prom::gfp* expression in *let-381(ns1023)* mutant. The lateral and ventral HMC processes are missing.

Development • Supplementary information

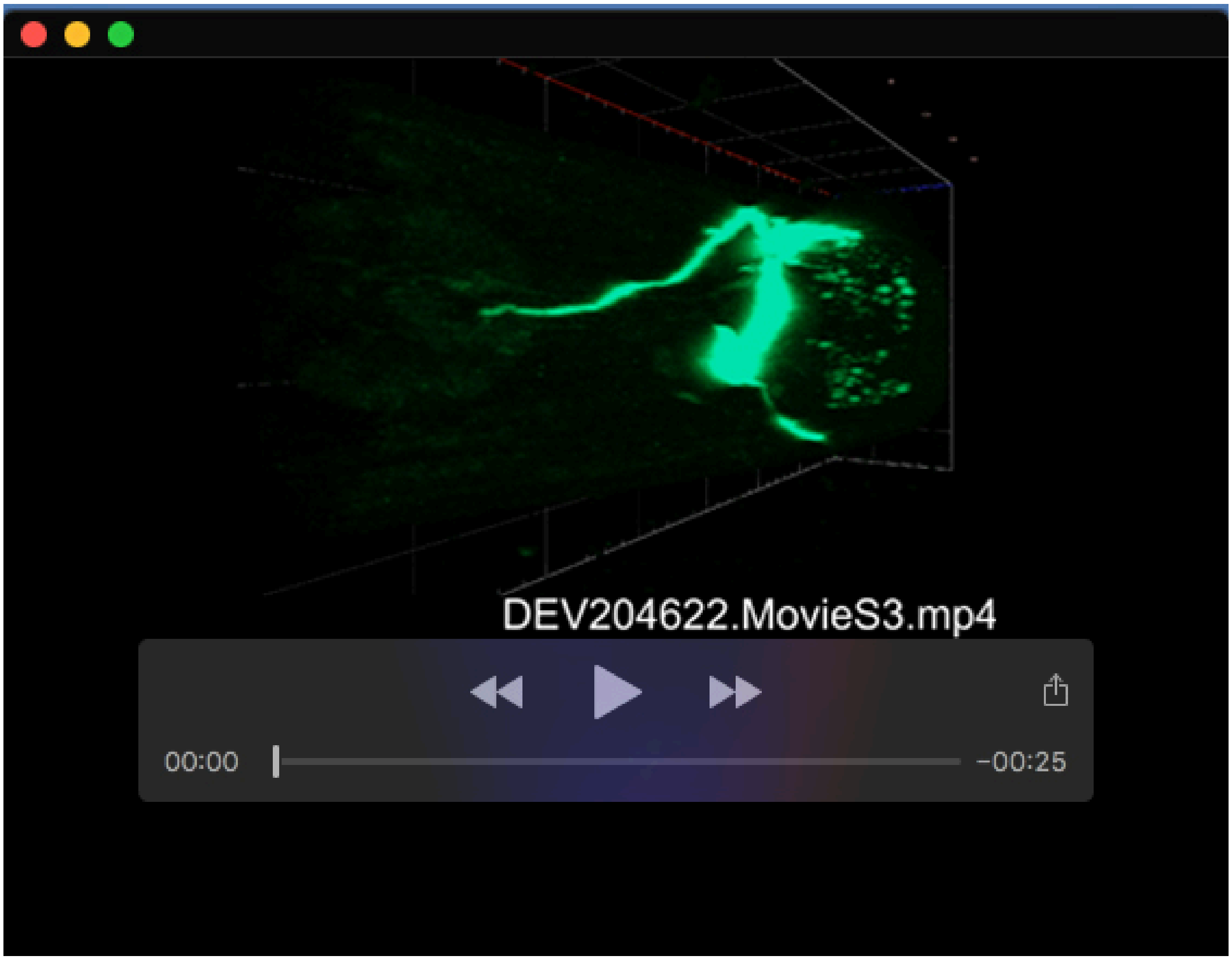

Movie 3. HMC with Defective Morphology in let-381(ns1023) mutant background, example 2. *arg-1prom::gfp* expression in *let-381(ns1023)* mutant. There is an anteriorly extending process and only one lateral process that reaches the ventral side. Ventral process is missing.

Development • Supplementary information

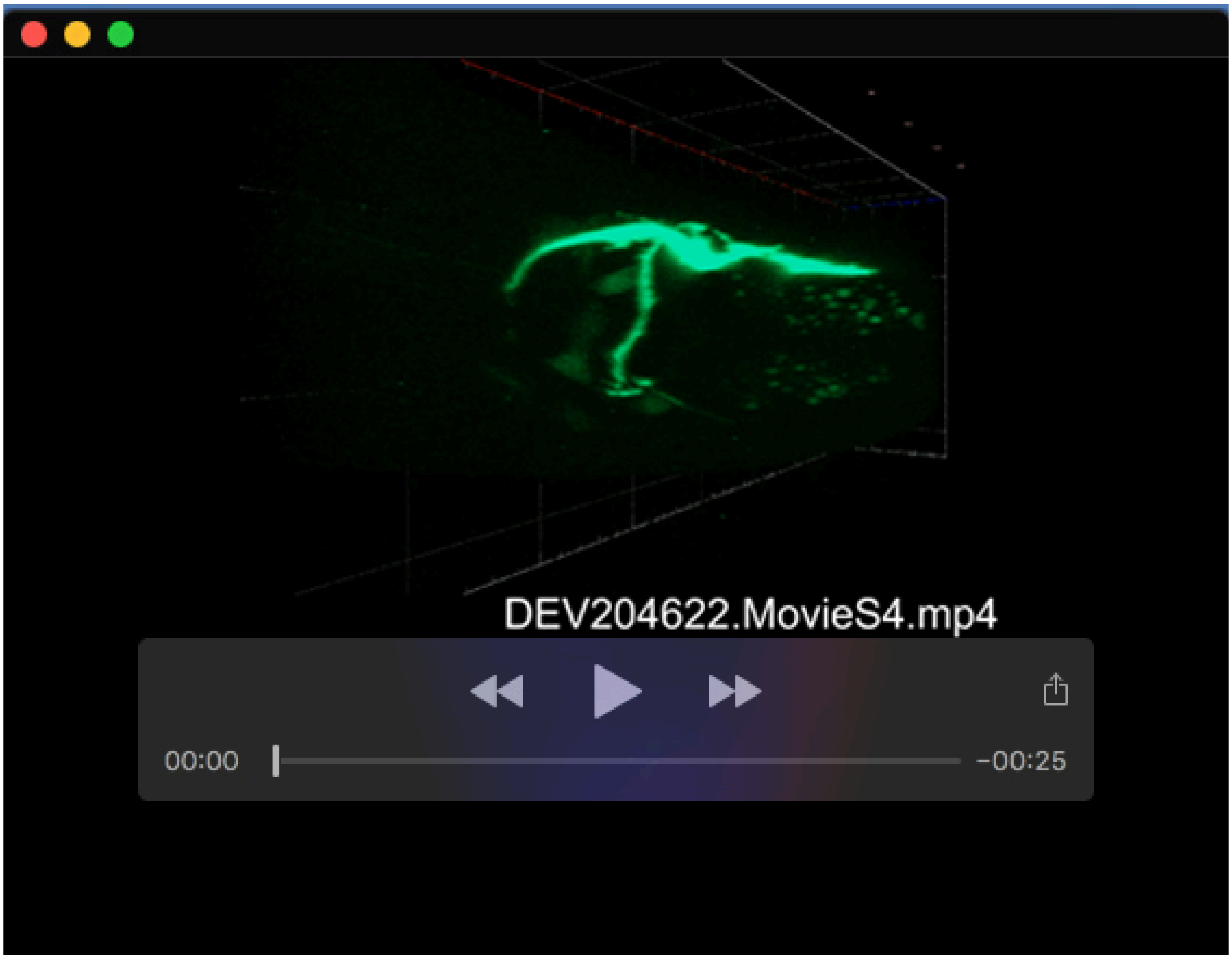

Movie 4. HMC with Defective Morphology in let-381(ns1023) mutant background, example 4. *arg-1prom::gfp* expression in *let-381(ns1023)* mutant. One of the two lateral processes is very short. Ventral process is missing.
